# Digital interventions targeting excessive substance use and substance use disorders: a comprehensive and systematic scoping review and bibliometric analysis

**DOI:** 10.3389/fpsyt.2024.1233888

**Published:** 2024-02-05

**Authors:** Magnus Johansson, Danilo Romero, Miriam Jakobson, Nelleke Heinemans, Philip Lindner

**Affiliations:** ^1^ Center for Psychiatry Research, Department of Clinical Neuroscience, Karolinska Institutet, & Stockholm Health Care Services, Region Stockholm, Stockholm, Sweden; ^2^ Center for Dependency Disorders, Stockholm Health Care Services, Region Stockholm, Stockholm, Sweden

**Keywords:** digital, eHealth, mHealth, iCBT, addiction, substance use (drugs, alcohol, smoking)

## Abstract

Addictive substances are prevalent world-wide, and their use presents a substantial and persistent public health problem. A wide range of digital interventions to decrease use and negative consequences thereof have been explored, differing in approach, theoretical grounding, use of specific technologies, and more. The current study was designed to comprehensively map the recent (2015-2022) extant literature in a systematic manner, and to identify neglected and emerging knowledge gaps. Four major databases (Medline, Web of Science Core Collection, and PsychInfo) were searched using database-specific search strategies, combining terms related to clinical presentation (alcohol, tobacco or other drug use), technology and aim. After deduplication, the remaining n=13,917 unique studies published were manually screened in two stages, leaving a final n=3,056 studies, the abstracts of which were subjected to a tailored coding scheme. Findings revealed an accelerating rate of publications in this field, with randomized trials being the most common study type. Several meta-analyses on the topic have now been published, revealing promising and robust effects. Digital interventions are being offered on numerous levels, from targeted prevention to specialized clinics. Detailed coding was at times made difficult by inconsistent use of specific terms, which has important implications for future meta-analyses. Moreover, we identify several gaps in the extant literature – few health economic assessments, unclear descriptions of interventions, weak meta-analytic support for some type of interventions, and limited research on many target groups, settings and new interventions like video calls, chatbots and artificial intelligence – that we argue are important to address in future research.

## 1 Introduction

Addictive substances – ranging from legal but typically controlled ones likes alcohol and tobacco, to illicit drugs like cocaine, as well as prescription opioids with high abuse potential – and the negative consequences of use thereof, are top-ranking contributors to the global burden of disease (1), by causing high societal costs (2) and decreased quality of life (3, 4). Public health initiatives to combat this include both different types of supply-side restrictions (5) as well as primary prevention efforts focused on reducing demand (6). In clinical settings, there are now several evidence-based pharmacological and psychotherapeutic treatments available for substance use disorders (7), yet success rates remain far from perfect and issues like treatment provision and perceived barriers to treatment-seeking (8) continue to result in a significant treatment gap (9). The now ubiquitous integration of consumer information and communication technologies into everyday life, constitutes an attractive vector to disseminate prevention and treatment interventions. Such digital interventions present unique benefits, including (in principle) unlimited dissemination potential at low cost (10); Big Data capabilities including machine learning outcome prediction (11); delivery of standardized yet adaptive evidence-based content (12); reduced barriers to treatment-seeking through increased availability and/or decreased stigma (13); possibility of integrating non-invasively collected data from wearables and similar (14); and more.

Digital screening and brief interventions (15), as well as discussion groups dedicated to excessive substance use (16) surfaced already in the 1980’s and since then, the field of digital interventions targeting addictive substance use and substance use disorders has grown exponentially. Although recent reviews and meta-analyses have surveyed specific types of interventions and/or substances (17, 18), we are not aware of any recent comprehensive and systematic review, that can provide an overview of the field at large. The purpose of the current study was to perform such a scoping review, with the aim of summarizing and coding recent (2015-2021) trends in digital treatment and prevention interventions: what type of research has been performed, for what substances (alcohol, tobacco, or other drugs) and using what technology. In doing so, we aimed to uncover and highlight key remaining and emerging knowledge gaps in the field, which may in turn serve to inform both new original research, and future systematic reviews and meta-analyses on delimited, previously neglected topics (19). In addition, in the style of a narrative review (20), we also include descriptive summary and synthesis of the findings of the many meta-analyses and systematic reviews that have been performed on the topic of efficacy.

## 2 Materials and methods

### 2.1 Search strategy

A systematic and comprehensive literature search was performed in collaboration with research librarians at the Karolinska Institute University Library. The search strategy covered inclusion and exclusion criteria were formulated according to both PICO (Population, Intervention, Comparison, Outcome) and PEO (Population, Exposure, Outcome) frameworks, see the **Supplementary Material** for details. Except for research focusing on humans, no restrictions regarding population or comparison were used. Intervention or Exposure was interventions aimed at preventing or reducing use, excessive use and addiction (abuse and/or dependence) to alcohol, narcotics, doping or tobacco, that are delivered digitally via computer, tablet, smart phone or equivalent. Outcomes included were outcomes that can be related to abstinence from use or reduced use of alcohol, narcotics, doping or tobacco. Outcomes related to experiences of or preferences regarding digital interventions were also included.

In the first stage, a test search was run to identify the relevant MeSH and free-text search terms, the results of which were compared to a set of known studies. Next, the preliminary search strategy was revised to increase sensitivity. The search was performed in three databases on 2021-11-19: Medline, Web of Science Core Collection, and PsychInfo. The database-specific search strategies were created by combining terms related to clinical presentation (e.g. “tobacco OR nicotine OR smoking”) with terms related to technology (e.g. “SMS OR social media OR web OR internet”) and study aim (e.g. “prevent OR treatment”). The initial search identified n=11,062 hits published before 2015 and n=17,053 hits published in 2015 or later. After using a validated method for deduplication (21), the search hits were reduced to n= 6,691 publications before 2015, and n=9,981 publications in/2015 or later. The search was re-run on 2023-01-23, at which time an additional= 3,936 publications were added. The final number of screened studies published 2015 or later, thus totaled n=13,917. See the **Supplementary Material** for exact search terms used with each database and number of resulting hits.

### 2.2 Extraction and coding

**Figure 1:**
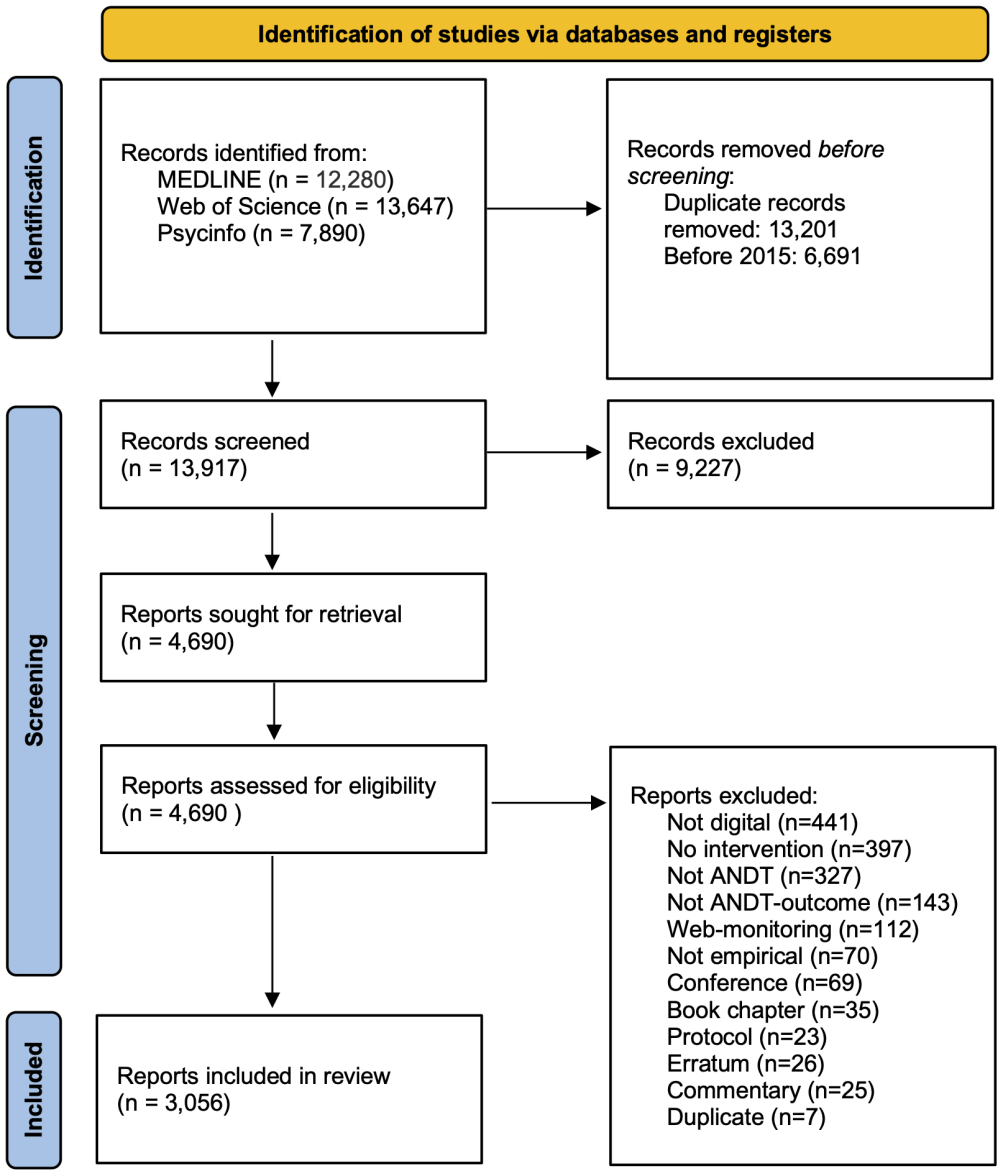
Flow chart.

See **Figure 1** for flow-chart. The final, deduplicated search results were imported into the Rayyan online software (www.rayyan.ai), which was used for subsequent processing. In the first stage, all n=13,917 records were assessed manually for initial topic eligibility by screening the title and (if needed) the abstract. A random sample of 600 titles were assessed by at least two of the authors who were blinded to each other. The assessment differed between authors regarding 50 titles and the differences were resolved in consensus discussions. This resulted in n=8,881 records being excluded for not being about interventions, the intervention not being digital or not targeting alcohol, narcotics, doping or tobacco. Examples of excluded publications covered research on electronic cigarettes (without any digital intervention), digital data collection (outside the context of a digital intervention), and on pharmaceutical systems. Inclusion and exclusion criteria used to assess eligibility can be found in the **Supplementary Material**.

In the second stage, the abstracts of the remaining n=4,690 records were screened in detail, resulting in a final sample subjected to coding. To ensure a directed analysis covering features of key clinical and public health importance, a deductive approach was used in which abstracts were coded with respect to five characteristics: type of study, type of technology, type of substance, theoretical grounding, and target group/arena. Within those pre-defined main categories, codes were generated inductively, to ensure that the codes represented the presumed diversity in the research field. If the information in the abstract was insufficient to code type of study, technology or substance, the full text was retrieved (n=169). All randomized trials were additionally coded for type of outcome measure and level of prevention. Level of prevention used pre-determined categories; 1. *universal* for interventions that prevent use or potentially harmful use of substances in populations, 2. *selective* for interventions that prevent harmful use in risk groups 3. *indicated* for interventions that prevent harm from use of substances among individuals at risk, 4. *treatment* for interventions that reduce harmful use or treat individuals who have developed substance use disorders (22). From identified meta-analyses and systematic reviews information about number of included studies and effects of digital interventions were extracted.

All authors participated in the coding. Codes were derived directly from the texts and every new code was communicated to the whole group via the Rayyan tool. Similar and conflicting codes were discussed in a dedicated chat and in regular meetings, thereby facilitating investigator triangulation (23). A random sample of 615 abstracts were coded by at least two of the authors separately and a preliminary coding scheme was created. Conflicts in the coding of 92 of these abstracts were discussed and resolved with all coders. In addition to the manual coding of each manuscript all identified codes, including variations, were additionally used as search terms in Rayyan to secure that all abstracts relevant for each code were identified. The most common codes in each category and some additional examples identified codes in each category are presented under results. A full list of codes and categories can be found in the **Supplementary Material**. The effects of digital interventions in each category as reported in included systematic reviews are summarized to highlight the results found in the most recent and most comprehensive reviews.

In addition to presenting descriptive statistics pertaining to the research questions, a bibliometric analysis was conducted, comparing the number of included publications included per year with the total number of publications per year in PubMed; these comparison numbers were retrieved using the easyPubMed R package (24).

## 3 Results

**Figure 2:**
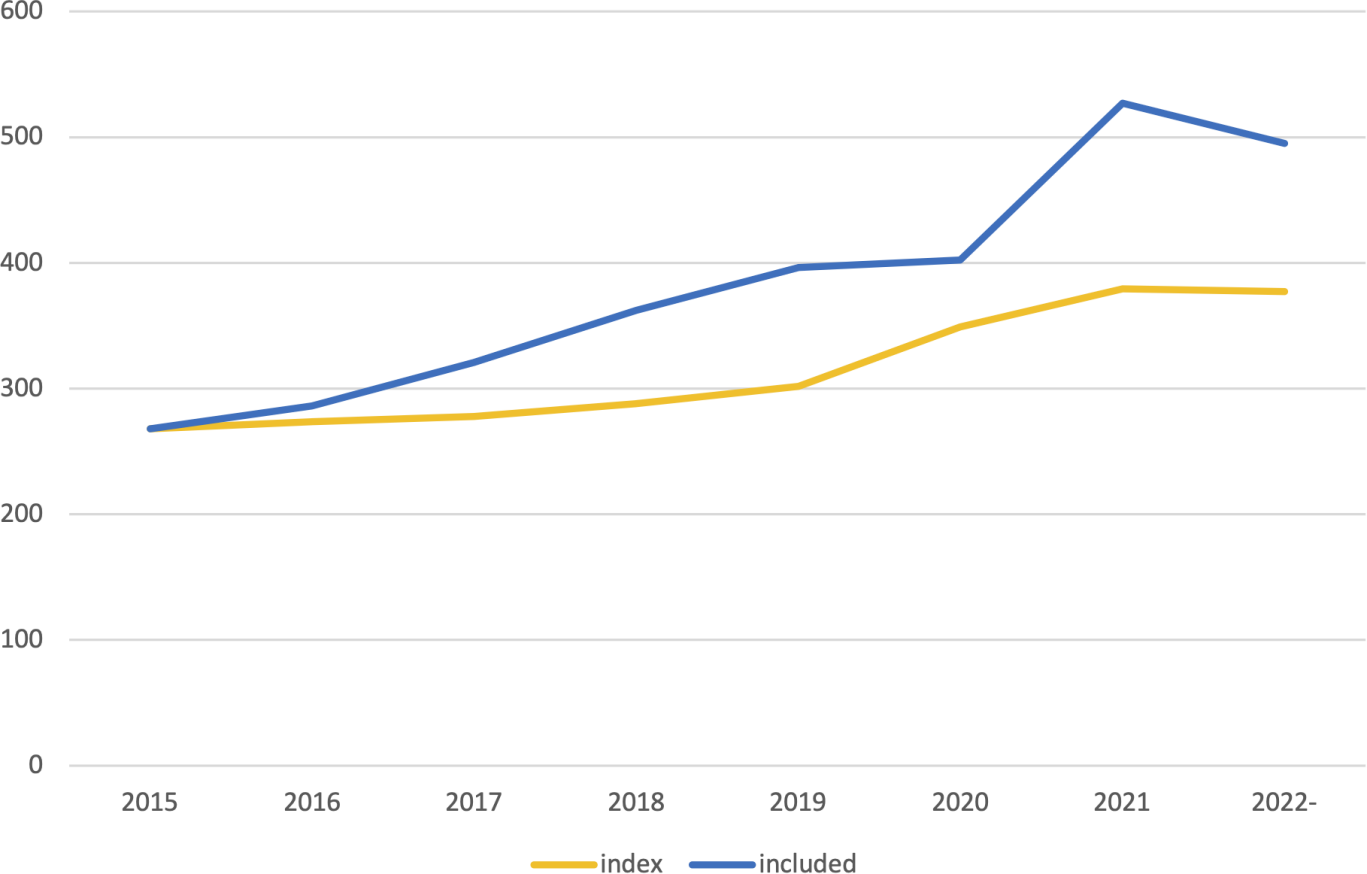
Included number of publications per year. Index was calculated based on the development in total number of publications per year in PubMed retrieved using the easyPubMed R package.

The final, coded sample of studies included n=3,056 publications. The number of publications on digital interventions for substance use included in this review increased by 227 (85%) between the year 2015 and the year 2020, corresponding to a linear, annual increase of 18.6 publications from 2015 through 2022. The included publications were published in 640 unique scientific journals. The most common focus of these journals were substance use/addiction and health/medicine. **Figure 2** shows the development in the number of articles included each year since 2015. **Table 1** shows the number of identified publications, included publications and unique journals of publication each year since 2015. **Table 2** shows the number of publications per journal category.

**Table 1:** Number of identified publications, included publications and journals of publication each year.

Year	Identified	Included	Journals
2015	1119	268	120
2016	1181	286	135
2017	1289	321	147
2018	1470	362	157
2019	1583	396	169
2020	1909	402	170
2021	2321	527	207
2022-	2096	495	194

**Table 2:** Number of journals and publications per journal category.

Journal category	Publications	Journals
Substance use or addiction	868	73
Health or medicine	808	296
Digital interventions	658	35
Psychology/psychiatry	308	132
Scientific methods	112	8
Technology	52	28
General	43	7
Pharma	33	14
Child/family	28	15
Other	19	12
Social work	15	7
Informatics	12	8
Prevention	8	4
Crime	7	6

### 3.1 Study design

The most common type of design was randomized studies (n=739). This category included randomized controlled trials, randomized clinical trials and randomized experiments. The randomized category also included pilot trials that were randomized (n=163), as well as a smaller number of factorial (n=33) and non-inferiority trials (n=6). The second most common category was development and testing of digital interventions (n=708), which include acceptability and/or feasibility (n=391), engagement (n=95) usability (n=44), and participatory-design (n=26) studies. The category quasi-experimental and observational studies (n= 344) included observational (n=94), quasi-experimental (n=74), pre-post (n=65), non-randomized pilot (n=146) and case-control (n=4) studies. Qualitative methodology was described in n=330 of the publications, including focus groups (n= 49), interviews (n=48), content analysis (n=45) and mixed methods (n=79). Secondary analyses of previously published trials was the topic of n=230 publications. Other types of designs included economical evaluations (n= 43), implementation studies (n=27) and case-studies (n=54). Of the included publications, n=374 were other reviews, of which n=172 were systematic reviews and n=68 meta-analyses. The search also identified several protocols for trials (n=305) and conference presentations, from which no findings had been published (n=140). See **Table 3** for the major study types per substance category.

**Table 3:** Type of study design and substance.

	All	Nicotine	Alcohol	Drugs	Opioid	Cannabis	Health behavior
**All**	3056	1192	1046	631	268	133	270
**Randomized**	739	306	319	128	30	47	58
	24,2%	25,7%	30,5%	20,2%	11,2%	35,3%	21,5%
**Reviews**	374	136	127	71	22	17	66
	12,2%	11,4%	12,1%	11,4%	8,2%	12,8%	24,4%
**Qualitative**	330	122	91	74	34	8	17
	10,8%	10,2%	8,7%	11,9%	12,7%	6,0%	6,3%
**Quasi-experimental**	388	160	111	97	54	17	23
	12,7%	13,4%	10,6%	15,4%	20,1%	12,8%	8,5%
**Development**	708	300	206	146	61	31	40
	23,2%	25,2%	19,7%	23,2%	22,8%	23,3%	14,8%

### 3.2 Technology

The terms used to describe the technology component of the digital interventions included “digital” (n=393), “electronic” (including “e-health”, n=378) or “technological” (including “tech”, n=62). The most common technology category was interventions delivered over the internet (n=1068), typically referred to via terms such as internet, online, or web (i.e., world wide web) interventions. Another common category was mobile interventions (n=795), which included smartphone apps typically native to iOS or Android. Other interventions were described as primarily text- based (n=423), usually delivered through SMS, email or other message services. Telehealth (n=370) includes concepts such as telemedicine and telepsychiatry, which usually involves healthcare contact via video or telephone, sometimes also combined with internet interventions, apps or text messages. In connection with the COVID-19 pandemic, telehealth and mobile interventions have become more common. Video conferencing was explicitly mentioned in n=65 of the studies. Computers or tablets (n=306) with installed applications or other offline-accessibility, were found to be have been used in healthcare or school settings.

**Figure 3:**
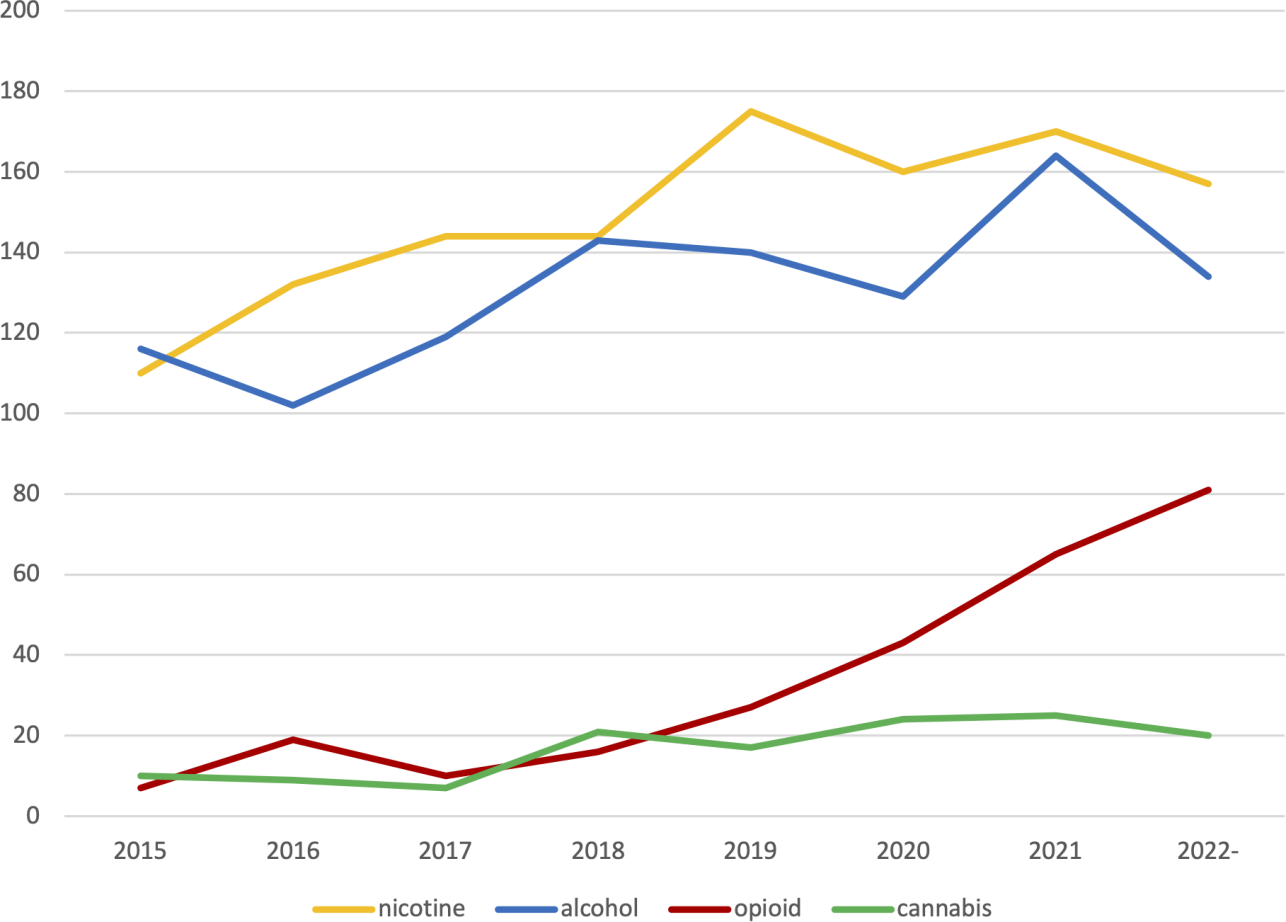
Number of publications per year on nicotine, alcohol, opioids and cannabis.

**Figure 4:**
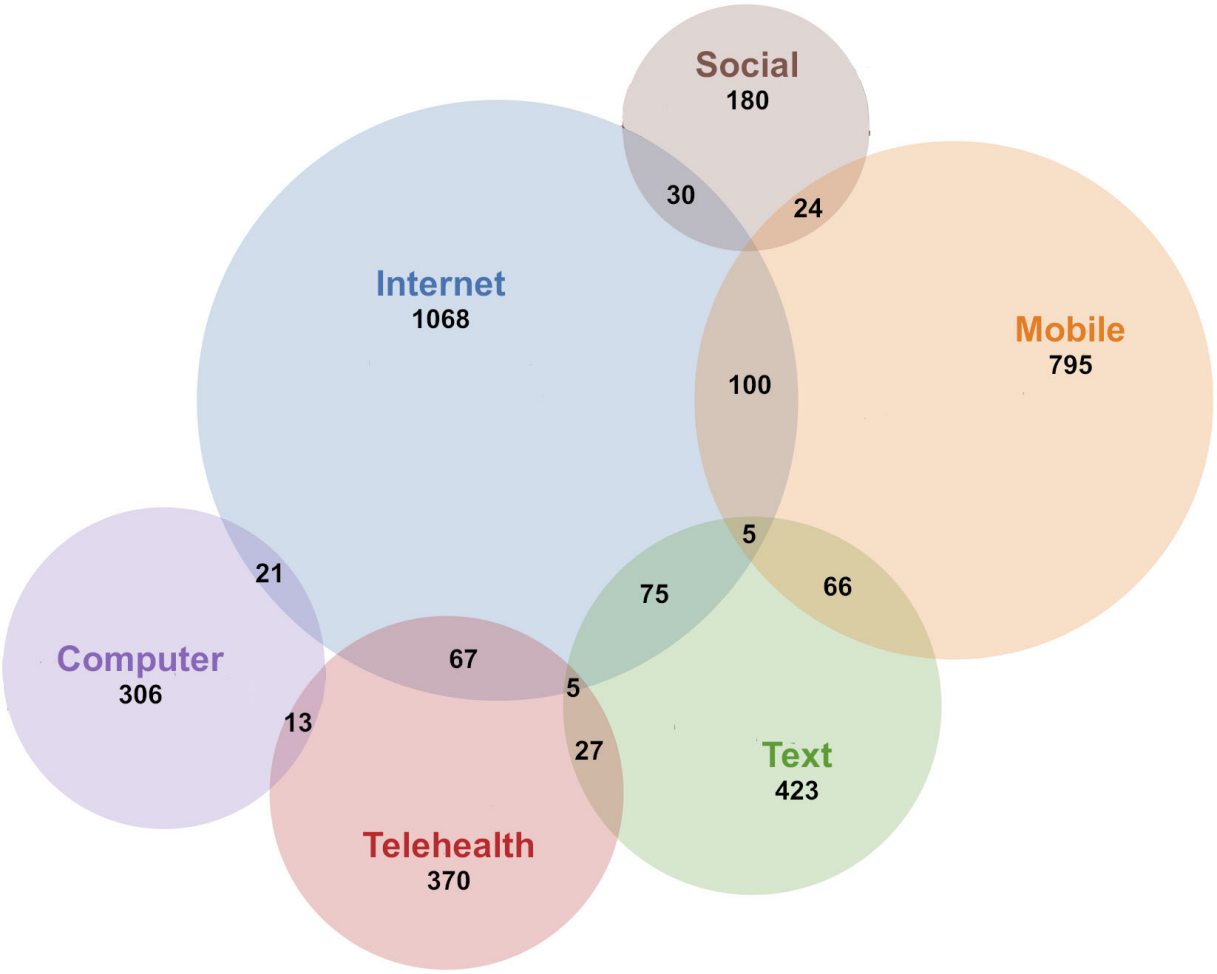
Overlap in use of technology.

Many studies combine several different technologies in the same intervention. Some included studies used so called blended interventions (n=181) that combine digital interventions with face-to-face contact. Others used digital interventions only as a smaller part of a larger intervention package (n=86). Comparisons between digital and face-to-face interventions were made in n=63 of the studies. Additional technologies covered included videos (n=64), virtual reality (VR, n=25) or gamification (the inclusion of game-like elements in non-game settings, n=57). A subset of studies featured wearable sensors (n=85). New ways of working via the internet also make it possible to be present where the intended target group already is; among the coded studies there were examples of using social media (n=180) and discussion forums (n=61). See **Table 4** for information on the different main categories of digital technologies that were found to have been used. The number of studies using different technologies per year is shown in **Figure 3**, while overlaps between different technologies is visualized in **Figure 4**.

**Table 4:** Type of technology used.

	All	Nicotine	Alcohol	Drugs	Randomized	Reviews	Qualitative
**All**	3056	1192	1046	631	739	374	330
**INTERNET**	1068	384	451	181	309	109	100
	34,9%	32,2%	43,1%	29,0%	41,8%	29,1%	30,3%
**MOBILE**	795	374	251	133	150	115	104
	26,0%	31,4%	24,0%	21,3%	20,3%	30,7%	31,5%
**TEXT**	423	239	132	52	148	24	34
	13,8%	20,1%	12,6%	8,3%	20,0%	6,4%	10,3%
**TELE**	370	152	49	122	77	35	33
	12,1%	12,8%	4,7%	19,6%	10,4%	9,4%	10,0%
**COMPUTER**	306	83	138	84	102	22	15
	10,0%	7,0%	13,2%	13,5%	13,8%	5,9%	4,5%
**SOCIAL MEDIA**	180	107	48	29	38	12	36
	5,9%	9,0%	4,6%	4,6%	5,1%	3,2%	10,9%

### 3.3 Substances

**Figure 5:**
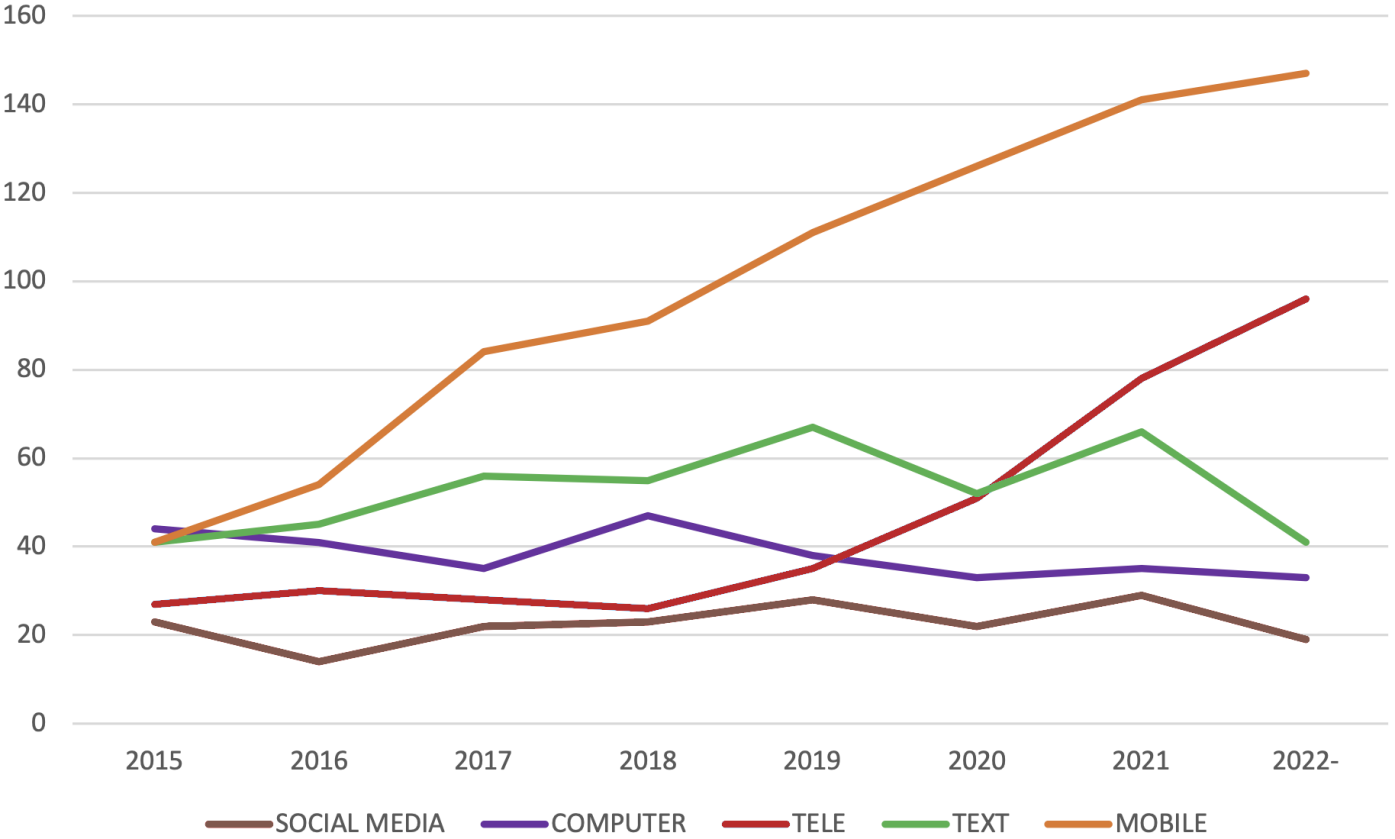
Number of publications per year using mobile, text, telehealth, computer and social media.

**Figure 6:**
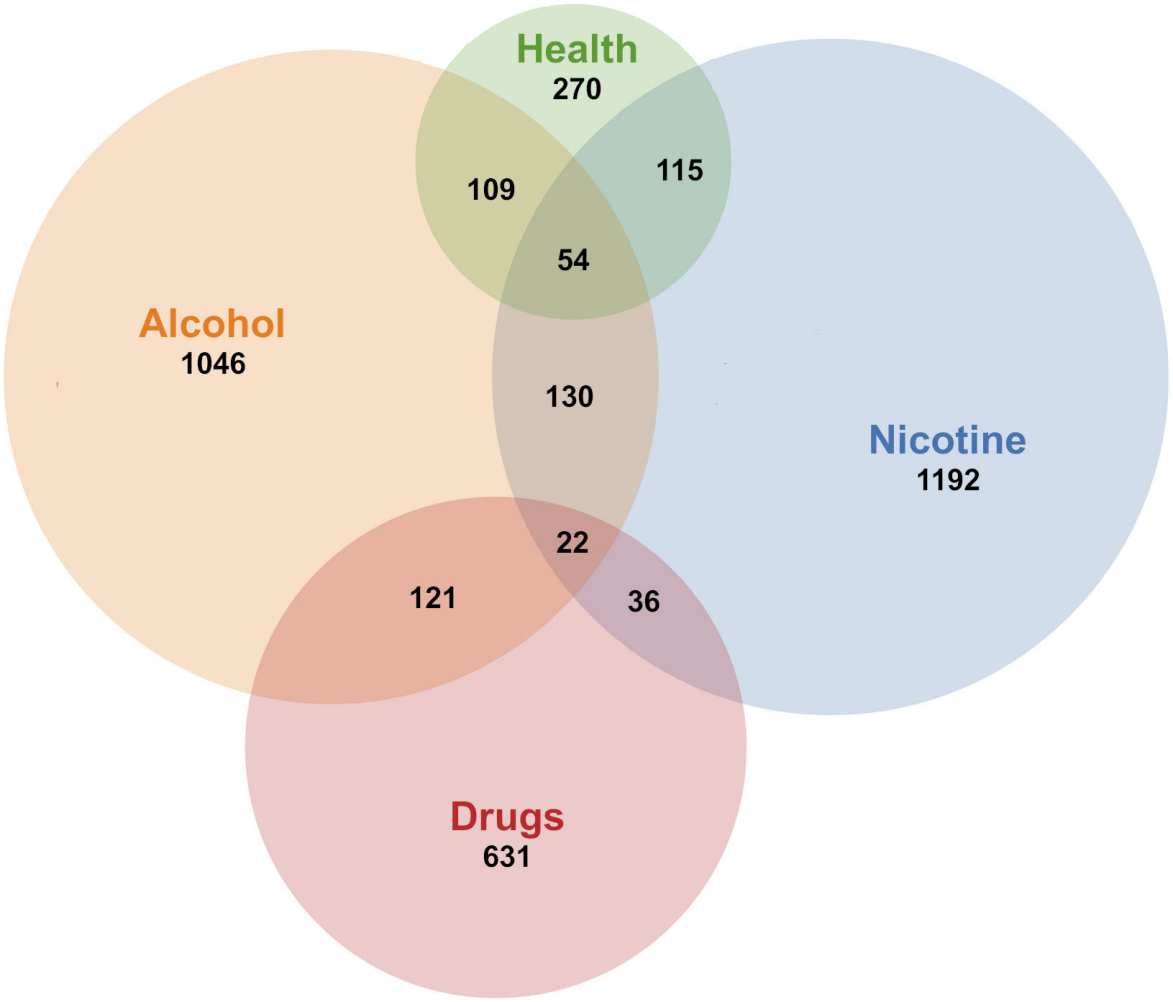
Overlap between substances.

The main substance categories identified were tobacco (n=1234), alcohol (n= 1156), and other drugs (n= 678). The category tobacco covered primarily publications about smoking (n=1054) or unspecified tobacco use (n=138), but also included vaping (e.g., electronic cigarettes, n=16), waterpipe (n=3) and smokeless tobacco (n=8). The drug category included all addictive substances that were not nicotine nor alcohol. The most frequent substances in the drug category were cannabis (n=133) and opioids (n=268). Other drugs covered were doping in sports (n=6), prescription medication (n=27), and illicit drugs (n=82), including stimulants (n=39). The category health-behavior (n=269) was created to collect studies that were not solely about substance use, but in which substance use was one focus or outcome, including.e.g. prescription drug use among users of a Behavioral Skills-Based Virtual Reality Program for Chronic Low Back Pain (25) or a review of electronic mental health interventions for Indigenous youth (26). In recent years, an increasing number of publications regarding opioids was observed. See **Figure 5** for the development of number of studies per substance each year and **Figure 6** for overlaps between substances.

### 3.4 Theoretical grounding

Theor ethical grounding of the studied intervention could be coded in n=2,016 (66%) of the included abstracts. The most common theoretical grounding was Cognitive Behavioral Therapy (CBT, n=271) which included e.g., community reinforcement approach (CRA, n=18), acceptance and commitment therapy (ACT, n=35). Another common category was brief interventions (n=279) which also included combinations with screening (SBI, n=99) and referral to treatment (SBIRT, n=43). The feedback category (n=177) included interventions using personal normative feedback (n=76) or feedback on blood alcohol concentration (n=41). Monitoring (n=109) included both self-monitoring (n=24) and ecological momentary assessment/interventions (EMA/EMI, n=38). The term Social interventions (n=160) was used to collect interventions with a prominent social component, like forums (n=61), social network (n=13) or peer support (n=70). Some interventions were based on different forms of cognitive training interventions (n=92), such as cognitive bias modification (n=56) or cue exposure (n=15). The Adherence to treatment category (n=208) included both referral (n=53) and adherence to different parts of treatment, e.g. medication (n=136). Contingency management which was the focus of n=45 studies, is used to both increase adherence to treatment and decrease substance use. Other mentioned theories used in the studies included the trans-theoretical model (also referred to as Stages of change, n=26), theory of planned behavior (n=23) and social cognitive theory (n=14). A few publications explicitly mentioned the use of so-called behavior change techniques, BCTs (n=28). **Table 5** shows the main categories of methods used in digital interventions.

**Table 5:** Intervention methods used.

	Nicotine	Alcohol	Drugs	Randomized	Reviews	Qualitative
**CBT**	61	112	74	81	13	16
**BRIEF**	24	200	54	116	8	22
**MI**	23	62	33	47	1	9
**Feedback**	18	148	17	97	12	12
**Cognitive training**	22	49	19	34	9	3
**Treatment adherence**	69	27	103	41	14	17
**Monitoring**	32	51	18	25	5	10

### 3.5 Target population and setting

The included articles describe digital interventions that target a wide range of populations; from the entire population of a country or speakers of a certain language to special risk groups or specific settings. The biggest special population targeted was health care (n=471) including conditions like cancer (n=43), cardiovascular (n=48), diabetes (n=14) or HIV (n=100) and settings like primary care and emergency care. A related category was mental health (n=202) that included e.g., depression (n=42), trauma (n=30) and psychosis (n=10). Young adults were categorized together with students (n=420) e.g., at college (n=126) or university (n=40). The category family included many different sub-categories; adolescents (n=223), parents (n=50), pregnant or post-partum (n=87) and concerned significant others of people with SUDs (n=48). Some digital interventions have been adapted to minorities (n=174) including different ethnic/cultural minorities, sexual and gender minorities (n=53) as well as people with disabilities (n=10). Digital interventions have also been tested among disadvantaged people (n=142) that also included individuals that are homeless (n=18), living in rural areas (n=39) or low- and middle-income countries (LMIC, n=22). SUD treatment and recovery were the focus of n=271 publications. This category also included methadone/buprenorphine substitution (n=57) and overdose prevention (n=28). Most interventions were aimed directly at individuals with hazardous or harmful substance use, but a smaller number of studies describe interventions that target whole groups or special settings. These include interventions in schools (n=36) or policy work among restaurants and bars (n=13). See **Table 6** for main categories of targeted populations.

**Table 6:** Groups or setting targeted.

	All	Nicotine	Alcohol	Drugs	Randomized	Reviews	Qualitative
**Student & young adults**	420	102	240	64	156	33	40
**Family & children**	307	89	108	70	78	36	38
**Healthcare**	468	248	163	84	128	34	44
**Psychiatry**	202	58	79	24	46	24	21
**Substance use treatment**	260	25	64	120	38	21	32
**Minority**	174	68	45	36	30	13	25
**Disadvantaged**	142	75	24	20	31	11	22
**Significant others**	49	9	17	12	17	2	10
**Professionals**	140	55	33	30	17	7	37

### 3.6 Level of prevention and outcome measures in randomized trials

Digital interventions for alcohol, tobacco and drugs have been studied in randomized trials at all different prevention levels: from primary, universal prevention (n=82) to tertiary interventions for people with substance use syndromes (including harmful use and dependence, n=104). The most common levels of prevention were indicated prevention for at-risk consumers (n=379) and selective prevention (n=178) for at-risk groups (e.g., students or people with various medical conditions that can be linked to the substances’ effects on physical and mental health).

Outcome measures in the randomized trials included in the overview were primarily various measures of substance use (n=367) or abstinence (n=153) that were measured by self-report or via biochemical measures such as exhalation or saliva, (n=48). Other measures used in studies where substance use was not relevant or possible to measure were e.g., intentions to use (n=44), attitudes (n=30) or knowledge (n=14) around using substances. In studies of the use of digital interventions, adherence were often used as outcome (n=62). In n=28 studies, substance use was only a secondary outcome measure. In some studies, the outcome measure was vaguely or unclearly described in the abstracts (n=37).

### 3.7 Narrative overview of the effects of digital interventions from systematic reviews

#### 3.7.1 Overall effects on use of different substances

According to a 2017 Cochrane review of digital interventions for *alcohol*, the effect compared with no or minimal interventions, is a reduction of 23 g (95% confidence interval (CI) -15 to -30) of alcohol consumed per week (27). Longer interventions appear to be more effective than short ones (12). A later meta-analysis of individual patient data demonstrated that internet interventions lead to an average reduction in weekly alcohol consumption corresponding to 50.2 standard units of alcohol (95% CI –75.7 to –24.8) and a higher rate of treatment response (odds ratio 2.20, 95% CI 1.63–2.95, *p* < 0.001) in comparison with various controls (17). Another Cochrane review found a significant effect of digital interventions on *tobacco* use compared to inactive control (Risk Ratio (RR) 1.15, 95% CI 1.01 to 1.30, n = 6786), but that the quality of the evidence was low (28). Another later meta-analysis on interventions targeting cannabis use showed that both digital prevention interventions (g = 0.33; 95% CI 0.13 to 0.54) and digital treatment programs (g = 0.12; 95% CI 0.02 to 0.22, p = 0.02) lead to reduced *cannabis* use compared to controls (29). Another meta-analysis (30) showed significant reductions after digital interventions in terms of use of *opioids* (g = 0.36; CI = 0.20 to 0.53) and *illicit drugs* (g = 0, 35; 95% CI = 0.24 to 0.45), but not of central stimulant drugs (4 studies, n = 481, P = 0.164).

#### 3.7.2 Effects using specific technologies

*Text messaging* for smoking cessation has been shown in a Cochrane review to be more effective as a minimal intervention (RR 1.54, 95% CI 1.19 to 2.00) and effective as an adjunct to other interventions (RR 1.59, 95% CI 1.09 to 2, 33; I2 = 0%, 4 studies, 997 participants), while smoking cessation apps showed no effect (RR 1.00, 95% CI 0.66 to 1.52) compared with minimal interventions (). Another review showed that digital smoking cessation results in higher smoking abstinence, both when delivered via web (Risk Ratio (RR) 2.03 (95% CI 1.7 to 2.03), as well as via mobile RR 1.71 (95% CI 1.35 to 2.16) or via text messages (RR 1.80, 95% CI 1.54 to 2,10) compared to inactive control groups (31).

The effects of mobile and text interventions to reduce the use of alcohol and other drugs remains unclear. A review of *mobile apps* showed that although most users reduced their use, less than a third of studies showed significantly better effects compared to comparisons (32). Regarding text message interventions, one meta-analysis showed that it is unclear whether they reduce weekly consumption (-18.62 g/week; 95% CI = -39.61 to 2.38) or heavy consumption of alcohol (-0.33 occasions/month; 95% CI = -0.79 to 0.12) and that the quality of the evidence was overall low (33). Moreover, few of the most common apps for substance use available in the prominent app stores have scientific support (34, 35) or use evidence-based methods (36).

The few reviews regarding video calls targeting substance use that have been conducted, have not been able to see any robust effects and suffer from methodological limitations. One overview suggests that video calls for substance use problems will likely positive effects, especially when other treatment options are lacking (37). According to a review study of video calls targeting various health-related behaviors that included four studies on tobacco and three on alcohol, only one study on tobacco showed significant effects of video calls in comparison to telephone calls (38). A scoping review of telemedicine for adherence in opioid treatment during COVID –19 showed limited evidence for similar outcomes compared to treatment as usual among the included studies (39).

A review of 13 studies of anti-smoking social media interventions found them to be effective in achieving smoking cessation compared to various controls (40). Interventions using cue exposure in virtual reality (41) for smoking cessation have so far shown inconclusive effects (42). Wearable sensors and other wireless technology can be used to monitor substance use or reduce the risk of overdose or relapse, but the effects of such interventions are still unclear (43). Moreover, results from a review of 10 studies suggest that chatbots can be used in psychiatric care (44).

#### 3.7.3 Effects of theoretical grounding

Digital *cognitive behavioral therapy* for alcohol has been shown to be effective in comparison with minimal control intervention (g = 0.20: 95% CI = 0.22 to 0.38) and as an adjunct to usual treatment (g = 0.30: 95% CI = 0.10 to 0.50) according to one targeted review (45). A review of *motivational interviewing* (MI) to reduce substance use shows that MI via telephone was effective and that positive effects for text and Internet-based MI have been observed in a smaller number of studies, for alcohol (46). *Personal normative feedback* appears to reduce drinking, both when used alone and together with other interventions, albeit with small effect sizes (47). Digital interventions to modify *cognitive bias* in users have thus far shown mixed results, without clear effects on substance use specifically (48, 49).

#### 3.7.4 Effects among specific groups

A review of preventive interventions targeting *parents* found that computer-based interventions were effective in reducing alcohol but not tobacco or drug use (50). A review of digital universal prevention primarily delivered in *schools* found significant but moderately-sized effects on alcohol or drug use outcomes of six youth programs (51). A meta-analysis of digital interventions in school that targeted changes in several health behaviors simultaneously showed no effects on alcohol or tobacco (52).

Digital interventions have been shown to reduce risky alcohol consumption among *adolescents and young adults* compared to assessment alone (53, 54). An updated review of tobacco interventions for young adults found “support for” text and telephone interventions (55). A meta-analysis of digital interventions for adolescents and young adults was not able to show that these led to significant reductions in cannabis consumption (56).

Digital interventions targeting *students* “can reduce” tobacco use according to one review (18). Meta-analyses have shown that digital interventions can produce a small but significant reduction in alcohol consumption among college students (57, 58). According to another review, digital interventions “can reduce” also the use of illegal drugs among students (59).

Among the elderly, digital interventions have shown “promising results” according to an overview (60). An review of digital interventions for women with substance problems showed that many studies have not assessed *gender-specific* effects and that support for specific effects among women is therefore weak (61).

One systematic review showed that digital interventions generally led to improvements in *concurrent problems with substance use and mental illness* compared to waiting lists or educational material (62). Another systematic review of digital interventions for co-occurring problems showed effects on both depression and substance use (63). A later meta-analysis showed mixed results with significant effects on depression at three but not at six-month follow-ups and on alcohol at six- but not three-month follow-ups (64). A review of tobacco interventions for people with serious mental health problems could not find any clear effect of digital interventions (65).

Digital interventions have been shown to improve behaviors that reduce the risk of *cardiovascular problems* (smoking, alcohol consumption and dietary habits combined with exercise) in the general population (66), but not among patients with cardiovascular disease (67). Digital interventions for *cancer survivors* increase the chance of smoking cessation, but support for achieving moderate alcohol consumption is still lacking (68). Digital interventions have also shown promising results in reducing substance use among pregnant women (OR=1.33, 95% CI 1.06 to 1.65, p=0.013) (69).

A review of digital interventions for alcohol in *primary care* was found to lead to reduced consumption or problems in most studies (17/24, 71%). In 13 out of 31 (42%) studies, better effects were also shown compared to treatment as usual. The involvement of healthcare staff and the use of implementation strategies were associated with better effects (70). Few studies have been conducted in *occupational settings* and a review showed only small and insignificant effects on alcohol consumption (71). There are various digital interventions developed to reduce substance use in *restaurant and pub* settings, but the effects of these are still uncertain (72). Digital training for professionals in providing support for smoking cessation has been shown in one review to be as effective as conventional training in knowledge and skills (73). Digital interventions that are mixed with traditional efforts (so-called *blended interventions*) have been shown to be able to increase efficiency, reduce dropouts and help patients maintain achieved changes in psychiatric care (74) and treatment for opiate addiction (75).

According to a review of digital interventions targeting people *recovering* from substance use disorders, just over half (55%) of 43 controlled studies showed positive results, with small or moderate effects, and just over half (57%) of 28 interventions showed positive effect in at least one study (76). There are also indications that digital interventions can be more cost-effective than treatment as usual in achieving abstinence among individuals with substance use disorders (77).

Reviews of digital interventions targeting *minority* populations have shown promising results in a number of reviews. Interventions targeting substance use and sexual health have been shown to be applicable and acceptable among men who have sex with men (78, 79). Preliminary effects of digital interventions have been shown on substance use among Hispanic and Black American minorities (80, 81). Digital interventions have been shown to be able to engage young people from indigenous populations (26) and half of the programs have shown positive effects on substance use (82). A review of digital interventions to reduce smoking among socially disadvantaged people showed significant effects up to 18 months (odds ratio 1.83, 95% CI 1.11 to 3.01) after the intervention (83).

## 4 Discussion

To our knowledge, this is the first comprehensive, systematic scoping review of the broader field of digital interventions for substance use and substance use disorders. By surveying relevant research and coding abstracts of studies published 2015-2022, we aimed to characterize the current state of the field and reveal remaining and emerging knowledge gaps suggestive of future directions for the field.

As evident by the number of publications in recent years, the width in terms of both targeted substance, methods and context, we conclude that the research field of digital interventions targeting addictive substance use is well-established and that it continues to grow. Indeed, more than half of all studies identified by our searches were published in or after 2015. A substantial number of protocols and conference proceedings indicates that more publications are on the way and the field is still rapidly evolving. This is particularly true for the subfields of mobile interventions and telehealth, that have seen the greatest increase in recent years. Others have found that the number of studies using social media to target substance use has increased between 2011 and 2017 (84), but a continued increase was not shown in our results. Another sign that the research area is well established is the number of unique journals, from different fields, that the papers included in this review have been published in. Bibliometric research has shown the growth of technology use in psychotherapeutic interventions overall (85).

The terms used to describe digital interventions covered by our review on substance use were similar to those found in neighboring fields (86). Surveying and summarizing this vast extant literature were made difficult by both varying terminology, technology developments and blended forms of interventions that have blurred the boundaries of what digital interventions are. Despite early attempts to establish a consistent use of terms describing digital interventions (87), these terminologies appear to have not been widely adopted. Importantly, we found no evidence that this heterogeneity appears to have decreased over time. Of note, initiatives like the CONSORT-EHEALTH reporting standard (88) were launched prior to the time period covered in the current study, yet do not appear to have had full impact yet. A recent Delphi study has highlighted several potential consequences of the inconsistency in terminology in digital interventions and suggested a possible common glossary (86).

Overall, a substantial number of meta-analyses show effects of digital interventions in the form of reduced substance use, that are similar to effects of face-to-face interventions, both in public health (27, 89) and specialized treatment settings (90). Compared to the smaller number of articles usually selected for meta-analyses, this review reveals a broader base of articles that use a range of different designs. Among the included publications, the most common type of design was randomized trials followed by different forms of studies of intervention development. However, there was a limited number of types of digital interventions that account for most of the research body and that also enjoy the strongest support: tobacco cessation via web or text (), computer or internet-based screening and brief intervention (91) and internet-based CBT (45) for alcohol, including for students, adolescents and young adults (54, 58).

Nonetheless, our scoping review revealed several knowledge gaps on several types and subcategories of digital interventions. Some interventions have not yet been studied enough, like digital interventions for illicit substance use (29). Others have shown inconclusive results, like mobile apps for alcohol use (32), or the use of social media and discussion forums for alcohol and drug interventions (92). Several common intervention components like cognitive bias modification and digital motivational interviewing (46, 48) have not yet been able to demonstrate robust convincing results. Moreover, there are promising but inconclusive results of digital interventions targeting specific groups e.g., older individuals, people with co-occurring problems, different minorities and schoolchildren (44, 60, 64, 71). Recent developments in technology and trends in use also highlight several emerging knowledge gaps. There is insufficient evidence on the effects of interventions based on videoconferencing (38), mobile sensors (43), virtual reality (42), chatbots (44) or artificial intelligence (93). Economic evaluations of digital interventions have shown promise, but more research is needed to demonstrate cost-effectiveness (77).

The categorization of randomized studies showed that digital interventions targeting substance use have been used on all levels, from universal prevention to specialized treatment. There was an emphasis in the included studies on indicated preventive efforts targeting individuals, rather than universal prevention, at risk groups or digital interventions aimed at people with substance use disorders. This may in part be explained by the search strategy which included intervention, as many universal prevention efforts may not be described as interventions.

### 4.1 Limitations

This scoping review has some obvious limitations. Congruent with the aim of a scoping review, the very broad scope and associated search strategy entailed that it was not feasible to code the entirety of the surveyed literature in detail. Many publications included only elementary information relevant to digital interventions in the abstract (e.g., specific technology used, theoretical grounding, outcome measures), lowering the level of detailed that could be compiled. For example, tobacco cessation interventions were mostly just described as smoking cessation without any additional information on the methodological or theoretical grounding for the intervention. A review also shows that only half of digital interventions for alcohol mention theory and even fewer used theory to select or develop the intervention (94). The broad scope also meant that quality assessment of the publications included was not possible. Even if many of the systematic reviews and meta-analysis included made their own quality assessments, the statements in the narrative overview of effects of digital interventions should be interpreted with some caution. Non-consistent use of terminology in most of the identified categories made detailed coding even more difficult. Some of the digital interventions included in the current review only used digital components for a small part of a larger intervention. This will likely become more common as digital interventions become increasingly integrated into regular health care settings. It is important to be able to distinguish these from interventions where the digital component is in focus or the main part of the intervention. One suggestion for a common glossary would be to start with a clearer threshold or definition on what constitutes a digital intervention.

### 4.2 Implications for future research

This scoping review has several implications for future research. The continued growth of the field revealed by our analysis highlights the need to make reference to recent publications. There is a need for updated reviews and meta-analysis both in general, and in different areas of digital interventions targeting different kinds of substance use among different populations. Many of the randomized studies identified in this review have not yet been included in meta-analysis. Moreover, many of the most recent general meta-analysis have mostly included studies published before 2015 (27–29). Since most of the randomized studies had use and/or abstinence as outcomes, there are good conditions for carrying out future meta-analyses. An obvious implication of the non-consistent use of terminology is that meta-analyses and systematic reviews on more targeted research questions need to continue having broad search term strategies covering a range of terms, to ensure total coverage. For example, now that HTML5 web applications have the same functionality as many smartphone applications and also can in parallel be accessed through traditional web browsers (95) – it would also be valuable to derive definition frameworks that accommodate these technological developments.

The evidence on some common interventions is still inconclusive (30, 32, 38) or even lacking on many newer types of interventions (42, 44), intervention components (46, 48), as well as target groups (44, 60), revealing a need for more randomized controlled trials of digital interventions. Hopefully the broad scope of the current review also can assist researchers in identifying areas with little or no previous research. In addition to the included articles, our review also identified work in the digital and substance use field that was not about interventions. One common such area was monitoring of social media and other web content or analyzed automatically with digital tools to detect trends in the use of, and attitudes towards substance use or compliance with regulations, e.g. online advertising and sales (96). Monitoring of online content, use of Big Data and AI raises many new ethical questions regarding digital interventions targeting substance use that have not yet been studied much (97, 98).

## 5 Conclusion

In conclusion, digital interventions targeting substance use constitutes an established field of research. Digital interventions in general seem to be effective in reducing substance use. Evidence on many of the more specific digital interventions are still lacking or inconclusive. The unveiled inconsistency in terminology to describe digital SUD interventions and their contents, may hinder synthetization of research findings. As the field is developing at a fast pace, there is a critical need for establishing a unified language in this area.

## Author contributions

MJ and PL designed the study, acquired funding, developed the search strategy and drafted the manuscript. MJ analyzed the extracted data and crated figures and tables. All authors contributed to the assessment of titles, extraction and coding, revising the manuscript draft and approved the final manuscript and are responsible for the content.
