# Supplementary material for: Digital interventions targeting excessive substance use and substance use disorders: a comprehensive and systematic scoping review and bibliometric analysis
Source: Front Psychiatry. 2024 Feb 5;15:1233888. doi: 10.3389/fpsyt.2024.1233888 (PMC10875034; doi:10.3389/fpsyt.2024.1233888)
Supplement: Supplementary file 1 [file Table_1.docx]

|  | **Inclusion criteria** | **Exclusion criteria** |
| --- | --- | --- |
| **Population** | Individuals, groups or populations. Directly to the target group, substance users or indirectly via professionals or relatives. | Not humans  Animal experiments, laboratory research or computer simulations |
| **Intervention** | Digital interventions (fully or partially delivered via Computer, Internet, Web, Telephone, Tablet, Mobile, Technology or Electronics) that are fully or partially aimed at preventing or reducing use, risky use and addiction to alcohol, narcotics, doping preparations or tobacco (ANTD) | Digital element of the intervention non-existent or minimal (e.g. simple reminders)  No intervention  surveillance or monitoring of web or social media  Not intended to influence substance use |
| **Comparison** | No limitation |  |
| **Outcome** | Abstinence or reduced use, risky use, addiction or harmful consequences of alcohol, drugs, doping preparations or tobacco  Knowledge, attitudes or intentions in relation to ANDT use  Use of digital interventions  Attitudes or intentions in relation to digital ANDT interventions  Experiences of, preferences regarding digital ANDT interventions.  Analyzes of content in digital ANDT interventions. | Not ANDT related  Not related to digital intervention |
| **Study design** | Empirical studies and reviews | Not empirical. Book chapters, comments, etc. that do not contain their own data.  Protocol for reviews  Conference presentations of data that have subsequently been presented |
| **Language** | No limitation | Abstract that could not be found in English or translated directly |
| **Publication date** | 2015 or later |  |

**PICO and PEO**

Population: All human populations

Intervention / Exposure: Interventions aimed at preventing or reducing use, risky use and addiction to alcohol, narcotics, doping or tobacco. Interventions that are delivered digitally via computer, tablet, smart phone or equivalent.

Comparison: No restrictions have been made based on the control group.

Outcome: Outcomes that can be related to abstinence from use or reduced use of alcohol, narcotics, doping or tobacco. Experiences of or preferences regarding digital interventions.
